# Developing Messages to Prevent Smokeless Tobacco and Nicotine Pouch Uptake Among Early Career Rural Firefighters in California: A Qualitative Study

**DOI:** 10.3390/bs16030470

**Published:** 2026-03-22

**Authors:** Roland Moore, Carol Cunradi, Katie Moose, Elizabeth Meza, Evi Hernandez, Raul Caetano

**Affiliations:** 1 Prevention Research Center, Pacific Institute for Research and Evaluation, 2030 Addison Street, Suite 410, Berkeley, CA 94704, USA; roland@pire.org (R.M.); rcaetano@pire.org (R.C.); 2 California Health Collaborative, 7311 Greenhaven Drive Ste. 270, Sacramento, CA 95831, USA; kmoose@healthcollaborative.org (K.M.); emeza@healthcollaborative.org (E.M.); ehernandez@healthcollaborative.org (E.H.)

**Keywords:** firefighters, smokeless tobacco, nicotine pouches, prevention messaging, workplace communication

## Abstract

This study describes participants’ views and insights into crafting effective communication aimed at smokeless tobacco and nicotine pouch prevention among fire academy trainees and new recruits. Firefighters have elevated rates of smokeless tobacco use compared with the general population. Nicotine pouches have also gained popularity among this occupational group. We launched a pilot project centered in rural Northern California counties to uncover factors that can be used to communicate smokeless tobacco and nicotine pouch prevention messages within the firefighter workplace. As a first step, we conducted semi-structured interviews with firefighter subject matter experts, including fire chiefs, fire academy instructors, wildlands firefighters, and recent fire academy graduates. This purposive sample (*n* = 13) was obtained through referrals from the project’s Community Advisory Board, composed of fire service professionals. Interviews were audio recorded and transcribed. Next, the qualitative interviews were thematically analyzed. The results focus on two aspects of effective workplace communication in the service to delivery of smokeless tobacco and nicotine pouch prevention messages: content (core information conveyed in a message), and format (how the message is transmitted or displayed). Examples of the former are the importance of keeping oneself healthy so that one can do one’s job; do not risk a future compensation claim due to smokeless tobacco or nicotine pouch use. Examples of the latter are the use of brevity; humor. Because firefighters often initiate use of these products after they join the fire service, communicating prevention messages in the workplace during the firefighter training and recruitment stage may help disrupt the uptake of nicotine products.

## 1. Introduction

Previous research has found that the rates of combustible tobacco use among firefighters were lower than the age-adjusted rates in the general population, yet rates of smokeless tobacco (SLT) use were elevated (Jitnarin et al., 2018; Jitnarin et al., 2015; Poston et al., 2012). An analysis of the 1992–2019 Tobacco Use Supplement to the Current Population Survey (TUS-CPS) found that while smoking prevalence declined overall, there was no change in the prevalence of SLT use among firefighters compared with non-first responders (Phan et al., 2022). Moreover, in 2018–2019 firefighters were more than three times as likely to use SLT than those in non-first responder occupations (adjusted odds ratio [AOR] = 3.4; 95% CI = 1.7–6.8; Phan et al., 2022). Based on these data, the prevalence of firefighter SLT use is estimated to be approximately 9%. In comparison, an analysis of the 2021 U.S. National Health Interview Survey found that SLT products were used by 2.1% of the general population (95% CI 1.9–2.4; Cornelius et al., 2023). When stratified by gender, the estimated SLT prevalence among men in the general population is 4.2% (95% CI 3.7–4.6; Cornelius et al., 2023), which is substantially lower than the estimated prevalence among firefighters, as noted above. Regarding women firefighters, a national survey found that the unadjusted rate of SLT use among the sample was 1.2% (Jitnarin et al., 2019). Based on age-standardized rates, women firefighters had similar rates of SLT use as women in the general population (0.5% vs. 0.3%) (Jitnarin et al., 2019).

Elevated SLT use by firefighters is of concern for several reasons. First, there is evidence that SLT increases the risk for all-cause mortality (Salazar et al., 2021). Second, SLT use may increase cancer risk because it contains more than 30 carcinogens (Cogliano et al., 2004; Richter et al., 2008; Sharma et al., 2022). For example, SLT is associated with an increased risk of head and neck cancer (Wyss et al., 2016). Given that firefighters are exposed to known carcinogens during fire suppression activities, which may increase the risk of certain malignancies (Daniels et al., 2015; Hwang et al., 2021), it is crucial to focus on modifiable risk factors such as SLT use. Climate change, and its attendant increase in the length and intensity of California’s fire season (Center for Climate and Energy Solutions, 2024), underscores the urgency of this task.

Overall use of tobacco, including SLT, is higher in California’s rural counties compared to its urban counties (California Department of Public Health & California Tobacco Prevention Program, 2025). Emerging tobacco products, such as nicotine pouches (NPs), have also gained traction in rural settings (California Department of Public Health & California Tobacco Prevention Program, 2025). Based on the Online California Adult Tobacco Survey, 2024, 1.6% of those aged 18–25 reported NP use, as did 2.2% of those aged 26–44 (California Department of Public Health & California Tobacco Prevention Program, 2025). NPs are marketed as a tobacco-free nicotine product (Delnevo et al., 2021; Robichaud et al., 2020). They are also marketed as a lower-risk, reduced-harm alternative to other tobacco products (Czaplicki et al., 2022; Hendlin et al., 2023). Tobacco control experts, however, note that NP uptake among young adults and new users can lead to nicotine dependence and potential use of traditional tobacco products (Keogh, 2021; Marynak et al., 2021; Stanfill et al., 2021). Moreover, NP products have not been on the market long enough to be subjected to analyses of the long-term consequences of use (Rungraungrayabkul et al., 2024).

To uncover factors that can be used to interrupt the initiation of SLT and NP use among new cohorts of rural California firefighters, we launched Firefighter Research About Nicotine and Tobacco In California (FRANTIC). Guided by community-based participatory research (CBPR) principles (e.g., true power sharing and collaboration in all phases of research) (Chung et al., 2010), the pilot project is a collaborative effort of tobacco-related occupational health researchers, project staff experienced in community tobacco control, and a Community Advisory Board (CAB) consisting of firefighters concerned about the health effects of these products.

The current study describes the thematic analysis of semi-structured interviews conducted with firefighter subject matter experts, in service of communicating SLT and NP prevention messages (via videos, study curricula, social media posts and workplace posters). The objective is to foster a change in social norms among rural firefighter trainees, early career firefighters, and their mentors/supervisors. Because a significant proportion of firefighters initiate SLT use after joining the fire service (Jitnarin et al., 2018), focusing on the firefighter training and recruitment stage may provide a window of opportunity to disrupt SLT and NP uptake. The purpose of this paper is to describe firefighters’ insights into how best to communicate such a prevention program to keep SLT and NPs out of the mouths of new firefighter trainees.

## 2. Materials and Methods

### 2.1. Community Advisory Board (CAB)

The FRANTIC project began as a collaborative partnership between tobacco-related occupational health researchers and community-based tobacco control advocates from two different non-profit agencies. The team launched a pilot project in 2023 aimed at disrupting initiation of SLT and NP use among new firefighters. Following a CBPR approach, project staff convened a CAB by inviting subject matter experts to participate in the project. CAB members include a rural county fire chief, rural county fire engineer, retired firefighter active in the Firefighter Cancer Support Network, metropolitan fire district health and fitness program manager, former hotshot (specially trained wildlands firefighter), and president of a large firefighter union.

### 2.2. Recruitment

Based on their contacts within the firefighter community, CAB members provided project staff with referrals for potential participants. The sampling strategy was purposeful rather than random to ensure the saturation of information about the details of firefighter training. The eligibility criteria were being aged 18 years or older; having specialized knowledge about the rural California firefighter trainee population due to their position as a firefighter professional, recent or current firefighter trainee, or firefighter program instructor; being an English speaker; and being physically and mentally able to participate in the interview. In recruiting for these interviews, tobacco and nicotine use were not an inclusion criterion. A $75 gift card was offered as a thank you incentive. Prior to the interviews, each participant provided written informed consent. The study protocol for the protection of human subjects was approved by the Institutional Review Board of Pacific Institute for Research and Evaluation, IRB ID# 2054993-3.

### 2.3. Interview Process

Between August 2023 and January 2024, two FRANTIC staff members conducted semi-structured interviews with 13 key informants to learn about rural California firefighter trainee exposure to, or involvement with, SLT and NP use, as well as the factors influencing these behaviors. An interview guide was developed using input from the CAB (See Supplementary Materials). Using a pre-determined set of open-ended questions, the interviews sought to elicit responses from the key informants on SLT/NP contexts of use in the fire service. Specifically, their insights were needed for the development of educational strategies and materials that can foster a change in social norms among rural firefighter trainees, entry-level firefighters, and their mentors/supervisors. The topics addressed included factors that may influence the uptake of SLT/NPs among firefighter trainees, the rural context of SLT/NP use, the role of firefighter occupational culture in promoting or inhibiting SLT/NP use, and potential avenues of prevention. We asked follow-up probes to encourage the respondents to elaborate on their answers. Due to the wide geographic spread of participants, interviews were conducted online using Microsoft Teams (with a duration of 45–60 min) and audio recording. For the current study, we focus on interviewees’ responses to questions on SLT/NP prevention messaging (Table 1).

### 2.4. Sample Characteristics

The sample consisted of nine men and four women. The work experience of the sample ranged widely, from those who had recently completed their firefighter academy training program to those with 30+ years of firefighter service. To illustrate this range, their specific job titles included: Chief, Captain, Engineer, Deputy Fire Marshal, Hotshot (specialized wildlands firefighter), Medic, Instructor, and Training Coordinator. In terms of race/ethnicity, male interviewees included one Native American, three white people, and one man who identified as multiethnic/multiracial; four males declined to state their race/ethnicity. Female interviewees included one Latina and three white people.

### 2.5. Analytical Process

NVivo Transcription was used to perform an initial transcription of the audio recordings; these transcripts were reviewed and edited for accuracy. Two researchers (the first and second authors) jointly read each interview and used Dedoose version 9.2.012 (Dedoose.com, 2024) to organize the thematic analysis of these transcripts. Whenever there was disagreement about the coding of a particular segment, discussion occurred about why differing themes were chosen until a consensus was reached. In particular, these discussions concerned consolidating overlapping codes. We then reviewed the entire range of codes, both a priori (e.g., gum disease concerns) and emergent (or unexpected themes such as the use of acronyms in training), and rearranged them into a series of major themes and minor subthemes. Thematic data saturation occurred after 10 interviews, as we heard very similar suggestions for effective content (e.g., cost savings and cancer presumption) and format themes (e.g., illustrating health consequences with graphic warnings) by the tenth interview, with no entirely new ideas for persuasive content or format in the proposed training focused on NP/SLT prevention for firefighter trainees.

## 3. Results

As noted in the introduction, respondents reinforced the shared understanding that cigarette use appears to be quite low among firefighters, the use of SLT has been prevalent for decades, and NPs have become increasingly popular in recent years. Moreover, some of the factors that interviewees identified with the uptake of these products include long hours, boredom, work stress, the need to stay awake and/or become alert when awakened for duty, social norms supporting use of SLT/NPs, rural cultural acceptance of these products, peer use, “fitting in”, and camaraderie. As a form of triangulation, it is worth noting that our CAB members asserted these same factors as the underlying reasons for the popularity of these products among young firefighters.

Of greatest relevance to the study goals of communicating workplace messages to prevent the uptake of SLT and NPs were the following major themes: why firefighters should not use tobacco, training techniques, and message reinforcement. Within these major themes, two primary aspects of prevention messaging emerged: content and format. We use the term message content to refer to the core information conveyed in the message. The term message format refers to how the message is transmitted or displayed. We provided illustrative quotes for message content and format to demonstrate the actionable elements of a brief, yet resonant curriculum identified by the respondents as a viable way to prevent young firefighters from initiating the use of these addictive products (see Table 2 for a list of these minor themes).

### 3.1. Prevention Message Content

Through thematic analysis of the interviews, we distilled eight core messages that we explicate below. The first four messages emphasize the centrality of firefighters’ maintaining their health on a long-term basis in order to fulfill their job demands in a sustainable manner. The next two messages focus upon the hierarchical culture of firefighters’ workplaces in the influential power of leaders and peers within the organization. The final two messages concern economic benefits resulting from avoiding SLT/NPs, both in the short and the long term.

#### 3.1.1. The Importance of Keeping Oneself Healthy So That One Can Do One’s Job

Physical fitness is essential for firefighters; the use of tobacco products runs counter to the goal of keeping oneself healthy. Variations on this theme arose in several interviews. For example, a long-term volunteer firefighter stated, “*…whether you’re a volunteer or not, you want to be a professional firefighter and your skills and abilities and you yourself are a tool… sense of duty to keep yourself healthy while you’re a firefighter, so that you can do your job right—and tobacco is counterproductive to that.*” By leveraging firefighters’ core values of physical health, this fundamental tenet underlies most of the following observations.

#### 3.1.2. It Is Unknown What the Long-Term Effects of NP Use Are—Do Not Be a Guinea Pig

Some participants brought up the unknown long-term effects of NP use. Incorporating this insight into advice for fellow firefighters, a recent trainee stated, “*if I use the nicotine pouches, it burns my gum. And I don’t know what I was doing in the long run because it’s only been two years. … I don’t want to be a guinea pig.*” This quote illustrates the healthy skepticism expressed by several of the interviewees, which could help stem the uptake of these products.

#### 3.1.3. SLT Has Been Shown to Increase the Risk of Oral Cancer and Gum Disease

In contrast to NPs, the harms of SLT are well-documented (Cogliano et al., 2004; Richter et al., 2008; Salazar et al., 2021; Sharma et al., 2022; Wyss et al., 2016). A hotshot identified gum disease as an incentive to stop using SLT, “*I do support stopping for health issues, like gum disease. Yeah, they [dentists] do the gum measuring tests. And, sometimes, on certain parts of my gum, it’s pretty bad where I usually have the dip. I’m assuming it eats away at my gums.*” This perspective echoes dentists’ warnings of avoiding oral tobacco products (Akers et al., 2006).

#### 3.1.4. Healthy Alternatives to SLT/NP Use Are Needed

In the poorly regulated market for over-the-counter remedies and supplements (e.g., nutraceuticals (Takefuji, 2025) and nootropics (Schifano et al., 2022)), there is a growing number of alternative products that some firefighters see as healthy alternatives to oral nicotine products. A medic suggested, “*Have you ever heard of grains where it’s the coffee pouches? And they’re like they’re like little chewing tobacco pouches, but they have coffee in them. And it’s kind of a way to not be using the nicotine and stuff like that and for the firefighters …to get that oral fixation of something kind of going on and still feel a little caffeine buzz.*” Firefighter interest in tobacco-alternative products is praiseworthy, but some of them (e.g., nootropic ones) may, at best, be untested, and at worst, may contain harmful materials that cause adverse reactions.

#### 3.1.5. Importance of SLT/NP-Free Role Modeling by Leaders

Firefighter leaders are emulated, particularly during the one-year probationary period in which they are attuned to the role-modeling of their supervisors at multiple levels (Revere, 2012). Some interviewees recognized the importance of leaders demonstrating nicotine-free behavior to their crews, particularly new recruits. A firefighter organizational leader and volunteer firefighter stated, “*Well, I would touch back again on mentorship. You know, intentionally or not, you will follow the behaviors and habits of those that are your mentors. If your instructor at the fire academy is, you know, has smokeless tobacco use, then there’s a really good chance that you’ll be susceptible to that.*” This perspective, however, requires the buy-in of firefighter leaders and mentors, some of whom use tobacco or nicotine products.

#### 3.1.6. Continue to Stay SLT/NP-Free Following Firefighter Training and Probation Periods

Several interviewees identified the challenge that trainees and new recruits face when exposed to the culture of tobacco and nicotine use in their workplace environments. A deputy fire marshal stated, “*And then at the end of the year, there’s usually …some type of a monster drill where there’s a written test and then there’s a massive physical test where you’re having 12 to 15 different evaluations and no one’s going to jeopardize their chances of passing that …using tobacco during that time frame. So I would say that …tobacco users are not going to be doing it during that time period*.” The challenge, however, is to remain tobacco- and nicotine-free following probation when surrounded by the physical and social availability of these products.

#### 3.1.7. Do Not Risk a Future Compensation Claim (Cancer Presumption) Due to SLT/NP Use

In 1982, California was the first state to adopt a cancer presumption law covering firefighters (California Professional Firefighters, 2025). Given the widespread occupational exposure of firefighters to potentially carcinogenic environmental toxins, the law presumes that any cancer that a firefighter is diagnosed with is attributable to these health hazards, unless the employer can prove otherwise. This concern was expressed by a CalFire instructor, “*That’s a tough one because, you know, if you go to the doctor and the doctor asks you, hey, you’ve got lung cancer, you know, is that exposed as well? You’ve also smoked for the last 15 years. How are you going to prove, you know, that it’s presumptive illness, in firefighting in the state of California now?*” Therefore, if a firefighter uses tobacco or nicotine products and is diagnosed with cancer, there is a risk that their compensation claim may be partially or fully denied based on the established association between tobacco use and numerous cancers.

#### 3.1.8. Consider How Much Money You Will Save over Time by Not Buying SLT/NP

The monetary cost of using SLT and NP can quickly add up. For example, the second author priced these products in October 2024 at a 7-Eleven located in Hanford (Kings County), California: a 15 NP can of ZYN 3 mg strength retailed for $6.99, and a 1.2 oz can of Skoal SLT retailed for $9.94. Some interviewees thought an effective prevention message would be to point out the cost of these products on young firefighters’ pocketbooks, and how the money could be used to save up for other purchases. For example, a CalFire Instructor said, “*I can’t believe the price of tobacco. The last 20 years, it’s gone through the roof. And, a pack of cigarettes cost as much as a case or a carton used to, back in the day.*” A hotshot observed, “*I’ve seen folks that have quit just because of the cost that they get. There’s some way to convey the price maybe over a year, you know. That could be effective …that you’re spending three grand a year.…*”

The interviewees identified a wide range of potentially persuasive arguments to avoid the uptake of nicotine pouches and chewing tobacco that could be communicated within the fire service workplace.

### 3.2. Prevention Message Format

Interviewees emphasized that the delivery and style of prevention messaging were just as important as the specific content of these messages. Accordingly, they identified the following salient aspects of effective communication aimed at firefighter trainees. The first five message format themes were identified as successful firefighter training techniques, whereas the sixth message format referenced an ineffective approach to avoid (condescending and age-inappropriate message framing). The final two message format themes identified appropriate messengers, including medical professionals, to deliver health information; young firefighter peers were prioritized as the most persuasive or memorable spokespersons if they could authentically convey their negative experiences with SLT/NP.

#### 3.2.1. Brevity

Some interviewees stated that SLT/NP prevention messages aimed at fire academy trainees should be brief. For example, a Deputy Fire Marshal noted that “*because they’re so focused on trying to just learn the fire essentials,*” it would be optimal for our curriculum to be “*short and to the point,*” especially because “*there’s so much that there’s not an hour in a fire academy that’s not accounted for.*”

#### 3.2.2. Humor

A Chief referred to the prevalence of morbid humor in the fire service and said that training required such humor for the information to be retained, “*Humor is really big because … a funny cartoon, a funny video, a funny joke, anything in there that … you get a laugh out of it, that’s going to stick with them.*”

#### 3.2.3. Graphic Images

Many interviewees stated that viewing graphic images depicting the harmful results of tobacco use would have a lasting impact. For example, a recent fire academy graduate said, “*I have cut back a tremendous amount because my mother saw fit to randomly assault me with a picture of a cancerous gum. … You can tell me about how this is bad for you. It’s going to go through one ear and out the other, but you can’t unsee a charred lung.*” Six of the 13 respondents urged us to use visuals to improve the chances of retaining the information and acting upon it.

#### 3.2.4. Hands-On, Interactive, Simulation

Although there is classroom instruction for firefighter trainees, much of their training takes place in interactive, hands-on settings. Some interviewees said that SLT/NP prevention messages would be more effective if there were a way to incorporate these characteristics in real-world settings. For example, a Captain said, “*I can talk about it and show you videos about it all day long, but until I actually put the nozzle in your hand and put pressure behind it, it’s a totally different world. So, hands on training is by far the best.*” However, it is uncertain how to translate this recommendation into action.

#### 3.2.5. For a Series of Facts, Consider an Acronym as a Reminder

The use of acronyms is an aspect of firefighter occupational culture (Prziborowski, 2006). Having a prevention message that uses an acronym may culturally resonate with firefighters. For example, a Chief said, “*I mean, there’s all these different things in there called the 18 watch out situations and the ten standard firefighting orders. If you made an acronym for tobacco that uses tobacco, something creative, even get firefighters’ input, so no smoke or something like that. And each letter would stand for something. And at the end of training, you go over the 18 watch out situations and you go over the ten standard firefighting orders and you go over the no smoke. That’s something that would stick…*”

#### 3.2.6. Avoid Talking Down or Condescension

Many fire academy trainees begin their training after graduating from high school; others begin as young adults in their twenties or as older second-career students. Given the audience of young men and women embarking upon life-saving careers, it is important that prevention messages impart respect and avoid any hint of condescension. For example, a fire organizational leader said, “*…Well, just all the basic rules of teaching: Don’t talk down. Don’t treat them like they’re stupid. And then don’t give them the one you’re going to give to the high school kids or … Don’t make it too simple.*”

#### 3.2.7. Health Messages from a Doctor

Some interviewees suggested that utilizing physicians, dentists, or other health care professionals to deliver SLT/NP prevention messages would increase their legitimacy and thus their effectiveness. A Captain said, “*Maybe couple that with a health care provider, a doctor, a physician, an oncologist, somebody like that.*”

#### 3.2.8. Peer Sharing Is Valued (e.g., Young Firefighters with Oral Cancer)

Firefighters view themselves as part of a brotherhood and sisterhood (Carter, 2021). Hearing an SLT or NP prevention message from a firefighter peer, especially one who used these products and received a cancer diagnosis, would carry gravitas. A Captain said, “*I think if you had other firemen maybe who went through a cancer scare in their mouth or jaw or throat, those are the guys that we would relate to the most.*”

## 4. Discussion

The sample’s purposively selected firefighter experts underscored the importance of both specific content and delivery style for communicating impactful public health messages about not initiating SLT or NP use. The timing of these messages is meaningful: once firefighters complete their initial training and are placed in settings where the use of these products is common, they need to have the initial awareness (via brief instructional videos, for example) of their hazards and require periodic reminders or boosters (e.g., social media or poster repetition of the original messaging), as the opportunities to convey these health messages are necessarily brief, in the face of many competing temporal demands. Themes identified by the study’s participants, such as health harms associated with SLT use, and the use of humor for effective prevention messaging, are in accord with findings from previous tobacco control research among young adults (e.g., Blanc & Brigaud, 2014; Wagner et al., 2019).

The results of this study should be considered in relation to behavioral theory. For example, study participants identified the importance of SLT/NP-free role modeling by leaders for prevention messaging. Social cognitive theory (SCT; Bandura, 1986) proposes a three-fold framework for acquiring new learning: modeling of recognizable peers; observing positive consequences for desired behaviors and negative outcomes for continued negative behaviors; and self-efficacy. Thus, if fire service leaders and peers model SLT/NP-free lifestyles to incoming firefighters, SCT suggests that this may help prevent the latter from initiating SLT/NP use. Similarly, study participants described perceived risks associated with SLT/NP use, such as oral cancer and gum disease. The health belief model (HBM; Glanz et al., 2015) identifies antecedents to health-related behavior and the factors associated with the likelihood of engaging in a behavior, such as perceived (health) threat; perceived benefits of engaging in a behavior; and perceived barriers to performing the behavior. The HBM would therefore provide a theory-based messaging framework to discourage uptake of SLT/NPs among new firefighters.

Previous qualitative research among firefighters has assessed barriers to SLT cessation (Jitnarin et al., 2021a) and cancer perceptions among SLT users (Jitnarin et al., 2020). Jitnarin et al. (Jitnarin et al., 2021b) conducted a beta test of a self-help SLT cessation program among a small number (*n* = 11) of firefighters; the program, “QUIT SPIT!” was based on a modified version of a previously developed and validated program, “Enough Snuff”. Their results supported the program’s feasibility and acceptability (Jitnarin et al., 2021b). To our knowledge, however, the current study is among the first to qualitatively explore how SLT/NP prevention message content and format can be crafted to discourage the uptake of tobacco and nicotine products among early career firefighters. Given the functional role that SLT/NP products play among this at-risk occupational group (e.g., camaraderie, the need to stay awake during non-standard shifts), developing and communicating effective prevention messaging in the workplace poses a challenge for occupational health and tobacco control researchers.

Examining tobacco control efforts in military populations may be instructive for SLT/NP prevention among firefighters due to certain occupational similarities between the two groups. For example, the fire service functions under a paramilitary structure, shares cultural traditions with the military, and has a disproportionate number of former military members among its ranks (Jitnarin et al., 2021b). Moreover, as with firefighters, rates of SLT among military personnel are elevated compared to the civilian population. In 2018, for example, 13.4% of active-duty personnel used SLT; in comparison, only 2.1% of U.S. adults reported SLT use (Meadows et al., 2021). Dunkle et al. examined factors associated with the initiation or re-initiation of SLT use among U.S. Air Force recruits (referred to as Airmen regardless of gender) following their mandatory tobacco-free basic military training and technical training (Dunkle et al., 2018). Their results showed that compared to never users, the strongest predictors of SLT initiation at one-year follow-up were male gender, with pre-basic military training use of cigarettes and cigars. Compared to former SLT users, the strongest predictors of re-initiation were male gender and intentions to use SLT. Lastly, compared to initiators of SLT, the strongest predictors of re-initiation were intentions to use SLT and peer use (Dunkle et al., 2018). Little et al. evaluated the impact of a brief tobacco intervention (BTI), a 40 min group-based intervention designed to reduce patterns of tobacco use among Airmen following their mandatory tobacco-free basic military training and the first two weeks of technical training (Little et al., 2020). Participants were randomized to one of three groups: BTI + Airman’s Guide to Remaining Tobacco Free (AG), (2) AG intervention, or (3) standard smoking cessation intervention. Although the BTI + AG condition did produce short-term changes in perceived harm, intentions to use tobacco, knowledge about tobacco products, and normative beliefs, there were not significant differences by condition in the use of tobacco products at the 3 month follow-up (Little et al., 2020). These findings suggest that implementing campaigns aimed at preventing the uptake of smokeless tobacco among those entering the fire service may also face similar challenges to those in military settings.

This study had several strengths. First, the study’s CBPR framework afforded project staff the opportunity to work in partnership with firefighter CAB members to develop a semi-structured interview guide. This helped ensure that the guide reflected on-the-job knowledge and concerns about SLT/NP use in the fire service that was culturally appropriate for this occupational group. The CAB was instrumental in identifying the key informants to be interviewed. Second, the study’s purposive sample, although small, represented a breadth of firefighter experience ranging from recent trainees to tenured fire chiefs, and included the voices of female firefighters. In terms of limitations, a larger sample size of respondents could have provided additional viewpoints, particularly from a greater number of early career firefighters who were fully immersed in the culture of firefighting several years past their training, as their perspectives would likely differ from those of the long-term career trainers who constituted the majority of our interviewees. As a pilot study, however, this set of interviews relied upon a purposively selected group of experts with lived experience, including both trainers and trainees whose widespread experience differences helped us to triangulate and to understand the nature of firefighter training, as context for the tobacco/nicotine product intervention we wished to create. Additional formative research with the firefighter target audience is necessary to develop appropriate SLT/NP interventions that resonate with the occupational culture.

Rather than relying on generic tobacco and nicotine product prevention messages, the results of this study can be used to develop and communicate prevention materials that can be tailored specifically for rural early career firefighters to dissuade them from initiating SLT/NP use. Given the increasing popularity of NPs, particularly in rural areas, there is an urgent need to effectively counter the allure of non-combustible tobacco and nicotine products whose use can result in nicotine dependence. Targeting at-risk occupational groups, such as early career firefighters, with SLT/NP prevention messages that integrate occupation-specific concerns and sensibilities may help disrupt the uptake of these products.

## Figures and Tables

**Table 1:** Interview questions on SLT/NP prevention messaging.

Potential Approaches to SLT Prevention What do you think would be the best approach to prevent SLT use among fire program trainees? If we were to offer a brief module for training programs that specifically shares prevention strategies to keep firefighters from initiating the use of tobacco/nicotine pouches or other nicotine products, at what point during the training and initiation process would be the best fit for it in the curriculum? What kind of content would you expect to see in a training module like that? Which instructional techniques would get the trainees’ attention in such a module? What would you recommend that we be sure to *include* in such a module, and why? What should we be sure to *exclude* from such a module, and why? How can the information shared in that module be reinforced in the first year or so of becoming a firefighter? What would get in the way of trainers using this tobacco prevention module? Conversely, what would encourage trainers to use this tobacco prevention module? To reinforce the messaging, what kinds of posters, digital education materials, social media, videos, or apps would you recommend for firefighter trainees? Although we have been focusing on firefighters who are not tobacco users when they come into the service, what about firefighters who come into the training program as smokers or smokeless tobacco users? What kind of messaging do they need?

**Table 2:** Relevant Minor Themes: Content and Format.

Prevention Message Content. The following prevention messages were gleaned from the interviews: Importance of keeping yourself healthy so one can do one’s job It is unknown what the long-term effects of NP use are—do not be a guinea pig SLT has been shown to increase the risk of oral cancers/gum disease Healthy alternatives to SLT/NP use are needed Importance of SLT/NP-free role modeling by leaders Continue to stay SLT/NP-free following firefighter training and probation period Do not risk a future compensation claim (cancer presumption) due to SLT/NP use Consider how much money you will save over time by not buying SLT/NP Prevention Message Format. Interviewees identified the following important aspects of educational materials: Brevity Humor Graphic images Hands-on, interactive, simulation For a series of facts, consider an acronym as a reminder Avoid talking down or condescension Health messages from a doctor Peer sharing is valued (e.g., young firefighter with oral cancer)

## Data Availability

The original contributions presented in this study are included in the article. Further inquiries can be directed to the corresponding author.

## Supplementary Materials

The following supporting information can be downloaded at: https://www.mdpi.com/article/10.3390/bs16030470/s1.
