# Supplementary material for: Developing Messages to Prevent Smokeless Tobacco and Nicotine Pouch Uptake Among Early Career Rural Firefighters in California: A Qualitative Study
Source: Behav Sci (Basel). 2026 Mar 22;16(3):470. doi: 10.3390/bs16030470 (PMC13024641; doi:10.3390/bs16030470)
Supplement: Supplementary file 1 [file behavsci-16-00470-s001.zip › behavsci-4101765-supplementary.pdf]

Firefighter SLT Semi Structured Interview Guide

This is a study about preventing smokeless tobacco use by rural California firefighter trainees. The study is being conducted as a partnership between the California Health Collaborative, a community-based organization, and PIRE California, Inc., a research organization; both organizations are non-profits. We hope to learn from firefighters, fire program instructors and mentors, and current or recent trainees why program trainees start using these products and how we can help prevent use. In all, 12 people will be interviewed.

Introductions

1. Tell us about your experience in the fire service (from screener). How long have you/did you work(ed) in this position?

2. Can you tell us about your personal experience with tobacco products? For example, have you, or someone you've known, ever used cigarettes or smokeless tobacco? If so, how long did they or you use these products? (If quit, ask about former tobacco use history and experience with quitting.)

Training Program Background

3. To start with, please describe the basic steps of a training program for firefighters: where and how are they trained? What big categories do they need to know to become a professional?

4. How do wildland, CAL FIRE, county, and city firefighter training programs differ?

5. How do firefighter training programs you know convey information via readings, lectures, discussions, simulations, or hands-on training, or some combination?

Training Program Tobacco Use Policy

6. Are there any policies or procedures governing the use of tobacco while in a firefighter training program?

7. Do you know of any policies regarding tobacco or nicotine product use while fighting fires, or in firehouses, or on the job generally?

8. Are there any policy changes that you would recommend to support reducing use of tobacco or nicotine products in the Fire Service?

Social and Physical Availability

9. In the training environment, and at fire stations, are there no smoking/vaping signs, or "smoke pits" (benches and ashtrays) set up for smokers near the training room, or other designated smoking/no smoking/vaping places?

10. Where do trainees typically obtain tobacco products? [convenience stores, or other places?]

11. What is your sense of the prevalence of smokeless tobacco or nicotine pouch use among trainees and new recruits? How about e-cigarettes? And cigarettes? Any other tobacco products?

12. Do you think that aspects of rural culture contribute to the uptake of tobacco or SLT among fire trainees? If so, tell us about those.

13. What aspects of firefighter occupational culture do you think may contribute to the uptake of tobacco or SLT among fire trainees? For example, do you think that using SLT is an unspoken way of "belonging"?

14. Do you think the transition from the fire academy to the fire service results in increased use of SLT? If so, tell us about that.

<u>Mental Health Issues and Other Substance Use</u>

15. In your organization's firefighter training program, how much attention is given to instruction in firefighter mental health (beyond safety in dangerous situations)? For example, coping with anxiety or depression?

16. How about training regarding alcohol or drug use?

17. Are there any training programs focusing on tobacco use?

<u>Potential Approaches to SLT Prevention</u>

18. What do you think would be the best approach to prevent SLT use among fire program trainees?

19. If we were to offer a brief module for training programs that specifically shares prevention strategies to keep firefighters from initiating the use of tobacco/nicotine pouches or other nicotine products, at what point during the training and initiation process would be the best fit for it in the curriculum?

20. What kind of content would you expect to see in a training module like that?

21. Which instructional techniques would get the trainees' attention in such a module?

22. What would you recommend that we be sure to *include* in such a module, and why?

23. What should we be sure to *exclude* from such a module, and why?

IRBNET ID 2054993-3 Approved Aug. 7, 2023

24. How can the information shared in that module be reinforced in the first year or so of becoming a firefighter?

25. What would get in the way of trainers using this tobacco prevention module?

26. Conversely, what would encourage trainers to use this tobacco prevention module?

27. To reinforce the messaging, what kinds of posters, digital education materials, social media, videos, or apps would you recommend for firefighter trainees?

28. Although we have been focusing on firefighters who are not tobacco users when they come into the service, what about firefighters who come into the training program as smokers or smokeless tobacco users? What kind of messaging do they need?

Anything else you want to share with us? We'd like to stay in touch with you and get your opinions/feedback/assessments as we develop the prevention materials. Would that be okay?

Thank you very much for sharing your thoughts with us; we very much appreciate it!

We would like to offer you a $75 gift card incentive in thanks for your contributions to this study.

IRBNET ID 2054993-3 Approved Aug. 7, 2023
